# An Epidemiological Study of Outbreak Investigation of Chickenpox in Remote Hamlets of a Tribal State in India

**DOI:** 10.7759/cureus.26454

**Published:** 2022-06-30

**Authors:** Anit Kujur, Kumari Asha Kiran, Manisha Kujur

**Affiliations:** 1 Community Medicine, Rajendra Institute of Medical Sciences, Ranchi, IND; 2 Preventive Medicine, Rajendra Institute of Medical Sciences, Ranchi, IND

**Keywords:** epidemiological study, vzv, gum boot epidemiology, outbreak, chicken pox

## Abstract

Background: Chickenpox is a benign, self-limiting disease caused by the varicella-zoster virus (VZV) that is transmitted from person to person with direct contact or airborne spread, which usually lasts for five to seven days. There was a sudden increase in the number of cases of fever along with rashes at two sites in Jharkhand, India. We aimed to survey and establish the etiology and investigate the extent of the disease.

Methods: We defined the case of chickenpox as a person with acute onset of diffuse maculo-papulovesicular rash. From the clinically suspected cases, blood samples were collected and tested for anti-VZV immunoglobulin M and immunoglobulin G antibodies (depending on the clinical features) with an enzyme-linked immunosorbent assay (ELISA) kit (Novatec Immundiagnostica GmbH, Dietzenbach, Germany). A detailed history was collected from each case including the history of contacts and immunization status.

Results: The outbreak investigations were done at two villages of the two different blocks and one school in the Bharno block. According to the case definition, 16 persons were found affected by the varicella-zoster/chickenpox infection who belonged to five different households of Itkhori village in Chatra district. The age group varied from four to 45 years. The mean age was 20.28 years. Out of 16 cases, 10 (62.5%) cases complained of fever, rashes, and itching, two (12.5%) reported rashes and fever, and four (25%) complained of itching along with rashes. While at the Bharno block of Gumla district, out of 62 cases that fulfilled the case definition, 55 (88.7%) cases complained of fever, rashes, and itching, two (3.2%) reported itching and rashes, one (1.6%) reported vomiting along with fever and rashes, one (1.6%) complained about pain and rashes, one (1.6%) complained of cough with rashes, and four (25%) complained of itching along with rashes. There was neither any death nor any serious complication noted due to varicella.

Conclusion: Chickenpox is still widespread in Northern parts of India like Jharkhand. Most of the cases were self-limiting and recovered at the Itkhori block, while at the Bharno block, there were 20 active cases and the rest were either recovered or were still in the recovery phase.

## Introduction

Chickenpox (varicella) is a highly contagious disease of childhood, but increasing reports of occurrence have also been reported amongst major chunks of adults. Chickenpox is a benign, self-limiting disease caused by the varicella-zoster virus (VZV) that is transmitted from person to person with direct contact or airborne spread, which usually lasts for five to seven days [1-4]. Persons with herpes zoster have an itchy and blister-like rash, and rashes are infectious during the vesicular stages with secondary attack rates in susceptible contacts of greater than 8.5% [5-7]. Chickenpox can also be serious and even life-threatening, especially in pregnant women and people with weak immune systems [3]. Vaccination is the only way to prevent it and is available for children [8,9]. Two doses of the chickenpox vaccine are given, and the vaccine is more than 90% effective in preventing chickenpox. The risk of getting chickenpox after completing two doses of chickenpox vaccine among the children is lower than after only one dose of chickenpox vaccine [3]. A decreasing trend of chickenpox in developed countries is due to vaccination, but developing countries like us still pose risk to be suffered from such diseases, as it is an optional vaccine in India. In the absence of vaccination, a progressive increase in seroprevalence with age has been found maximum in adolescents and adults [10,11]. Several outbreaks of chickenpox have been reported in different parts of the world and India from time to time [12]. A similar outbreak was reported from remote villages of Chatra and Gumla district of Jharkhand state where there was a clustering of cases having a similar presentation. Primary care physicians and grassroots level workers might play a critical role in providing accurate and timely information to health authorities, allowing for early intervention and outbreak management. This epidemiological investigation was done to provide expert opinion by conducting an epidemiological study to determine the characteristics of the outbreak, describe its source, identify challenges in case management, and suggest further course of action with recommendations for avoiding such outbreaks in the future.

## Materials and methods

An outbreak investigation for VZV was carried out on May 4, 2022, at two different outbreak sites in the state of Jharkhand, India. The two sites were the Itkhori block of Chatra district and the Bharno block of Gumla district. The state investigation team comprised various subject experts from Rajendra Institute of Medical Sciences (RIMS), Ranchi, one of the premier medical institutes of Jharkhand. The investigation team of epidemic surveillance staff consisted of doctors, research scientists, research assistants, and technicians for covering the fieldwork.

The outbreak investigation was carried out after the reporting of the outbreak from the district rapid response team to the state. The team was escorted by the local doctors of the concerned community health center, sahiya, auxiliary nurse midwife (ANM), and community members of that village to the houses of the reported cases. In addition to the visit to the reported cases, a door-to-door survey was also conducted to identify any unreported cases. For schools, the administrators were informed and active case detection was carried out throughout the school by the investigation team.

The investigation was carried out as a field study in which the team followed the standard principles of outbreak investigation for varicella as recommended by the Centers for Disease Control and Prevention (CDC). We defined the suspected case as per the CDC's probable case definition for varicella/chickenpox as an illness with acute onset of diffuse (generalized) maculopapular rash, without any apparent cause [3]. Therefore, as per the standard guidelines and probable case definition, the team carried out the investigation. This included the observational visit to the village where the outbreak occurred and the surrounding houses around the reported cases.

Based on the guidelines, an epidemiological sheet was prepared for the investigation by the team. Key interviews on the various aspects related to varicella/chickenpox infection were done with the people infected, their exposed contacts, their family members, and also with the service providers of that village who were directly or indirectly involved in the healthcare services in that area after obtaining written informed consent and in case of children less than 18 years of age, consent was taken from guardian/parent of each case. Approximately 5 ml of blood samples were collected and were labeled properly including name, age in years, gender, and address. All the samples were brought to the virology laboratory of RIMS, Ranchi by maintaining a cold chain. The serum samples were tested by the research scientist for anti-VZV immunoglobulin M (IgM) and immunoglobulin G (IgG) antibodies (depending upon the clinical features) by a commercially available enzyme-linked immunosorbent assay (ELISA) kit (Novatec Immundiagnostica GmbH, Dietzenbach, Germany), according to the manufacturer’s instructions. A sample was considered to be positive if the absorbance value was found to be more than 10% over the cut-off value. Similarly, the samples were considered to be negative if the absorbance value was lower than 10% below the cut-off value, while samples having absorbance values between 10% above or below the cut-off were considered to be in the grey zone. For all the ELISA kits that were used for diagnosis, the sensitivity and specificity of the test were >95% as per the manufacturer. For cases that tested positive for anti-VZV IgM, a convalescent serum sample was collected approximately 15 days after the first sample taken by the district response team of the respective district, since the CDC states that the experience with anti-VZV IgM antibody testing is limited and therefore it requires demonstration of rising titers of anti-VZV IgG for confirmation of chickenpox. In these cases, additional information was obtained on the number of lesions (<50, 50-99, 100-249, 250-499, or >500) to know the severity of the disease, other signs and symptoms (e.g. fever, itching, headache, body ache, and anorexia), history of comorbidities (e.g. asthma), and regular medicine use; information about chickenpox-related hospitalizations or complications (e.g. cellulitis, pneumonia, and encephalitis) was also obtained as per the CDC guidelines [3]. A vaccination card was also referred in each case for confirming the history of vaccination if available to know the vaccination status of each case.

## Results

The outbreak investigation was done at two villages in two different blocks and one school in the Bharno block. These outbreaks were not related to each other epidemiologically/geographically; hence, each outbreak was dealt with individually by two different teams.

Varicella-zoster outbreak investigation at Itkhori block of Chatra district

As per our investigation, the first case that fulfilled the case definition of varicella was a 45-year-old male who got the infection around the 10th of April 2022 in one of the remote villages named Pitiz under the Itkhori block. The village was approximately 11.8 km from the community health center (CHC) of Itkhori, Chatra district. According to the case definition, 16 persons were found affected by the varicella-zoster/chickenpox infection who belonged to five different households in that village. At the time of investigation, all persons were completely recovered and there were no active cases in that village.

**Figure 1:**
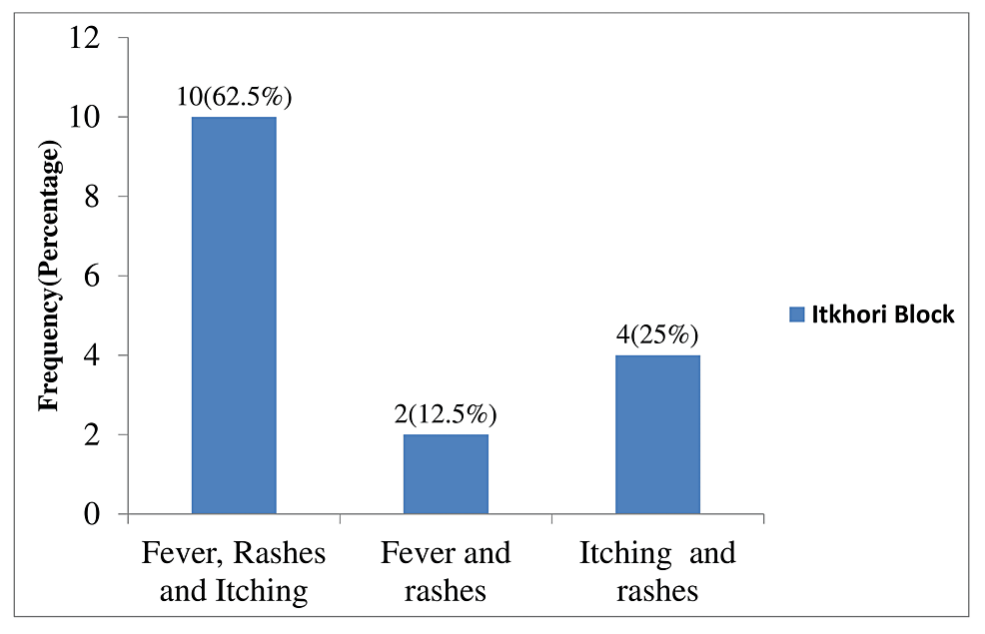
Clinical features among cases at Itkhori block

We interviewed the first case who was reported by the district response team. The first case of that village was a male of 45 years. He was a daily wage worker and frequently visited other places for work. His first rash appeared around the 10th of April 2022 on the chest, and then back, followed by limbs and face. As per his information, rashes were more than 50 in number. He did not seek any treatment from the nearest health center. His vaccination status was unknown. According to him, the duration of rashes was around nine to 10 days. Along with rashes, he got on and off fever and itching all over the body. He was not suffering from any other diseases and was not on any immunosuppressive drugs. He could not recall any potential exposure to varicella-zoster infection in the weeks before the onset of rash, as he frequently visits different places for his work. The second case of that village that appeared was also among his family. A total of four family members got infected with the same rash along with itching and fever. A total of 15 persons were fulfilling the case definition of varicella infection and were infected after the index case (Table 1). The age group varied from four to 45 years. The mean age was 20.28 years. None of them were vaccinated for routine vaccination of varicella-zoster/chickenpox infection. The mean duration of rashes was about nine days. Among all cases, macula-papular rashes occurred with itching all over the body. Most of them had rashes over the face, trunk, and limbs and none of the cases reported rashes on buccal mucosa. Out of 16 cases, 10 (62.5%) cases complained of fever, rashes, and itching, two (12.5%) complained of fever with rashes, and four (25%) complained of itching along with rashes (Figure 1).

**Table 1:** Outbreak investigation report at Itkhori, Chatra district

Serial number	Age in years/gender	Village	Date of onset of rash	Duration	Location of rash	Severity	Other symptoms	Vaccination status	Transmission setting	Household	Treatment taken
1	45/M (index case)	Pitiz	Around 10th April	9-10 days	All over the body except the palm and sole	Yes	Fever, itching	Unknown	Unknown	1	No
2	25/F (second case)	Pitiz	4-5 days after exposure to the index case	9 days	Chest, back, and limbs	Yes	Fever, itching	Unknown	Home	1	Yes
3	35/F	Pitiz	7 days after the index case	9 days	Face and leg	No	Fever	Unknown	Home	1	No
4	8/M	Pitiz	7 days after the index case	9 days	Hand and feet	No	Fever, itching	Not taken	Home	1	No
5	30/M	Pitiz	Around 16th April	11 days	All over the body	Yes	Fever, Itching	Unknown	Home	2	No
6	18/F	Pitiz	Around 16th April	9 days	Face	No	Itching	Not taken	Home	2	No
7	16/F	Pitiz	Around 16th April	9 days	Both hands and trunk	No	Itching	Not taken	Home	2	No
8	20/M	Pitiz	Around 17th April	9 days	All over the body except the palm and sole	Yes	Fever, itching	Not taken	Home	2	No
9	32/F	Pitiz	Around 17th April	9 days	All over the body except the palm and sole	Yes	Fever, itching	Unknown	Home	3	No
10	10/M	Pitiz	Around 17th April	9 days	All over the body except the palm and sole	Yes	Fever, itching	Not taken	Home	3	No
11	8/F	Pitiz	Around 17th April	9 days	All over the body except the palm and sole	Yes	Fever, itching	Not taken	Home	3	No
12	6/M	Pitiz	Around 17th April	9 days	All over the body except the palm and sole	Yes	Fever, itching	Not taken	Home	3	No
13	9/M	Pitiz	Around 16th April	9 days	Face, limbs	No	Itching	Not taken	Home	4	No
14	30/M	Pitiz	Around 16th April	9 days	All over the body except the palm and sole	Yes	Fever, itching	Unknown	Home	4	No
15	28/F	Pitiz	Around 16th April	9 days	Chest, back, limbs	No	Itching	Unknown	Home	4	No
16	4.5/F	Pitiz	Around 17th April	9 days	Chest, limbs	No	Fever	Not taken	Home	5	No

Only one case among the total cases visited the health center for treatment during the illness. As infected cases believed that illness was a state of presence of the goddess in their bodies, they believed that if this illness was treated with modern medicine, the goddess will get angry and they will get severely ill. Hence, this was the reason that most of them were not seeking healthcare advice. All the cases were applying ghee over the rashes and were using leaves of neem/Indian lilac to get relief from itching.

None of the cases infected from the illness were suffering from any other diseases like asthma, cystic fibrosis, cancer, and HIV/AIDS. None of the cases were vaccinated with the varicella-zoster/chickenpox vaccine.

Since the first case was reported on 10th April and the incubation period of the varicella-zoster infection was found to be 9-10 days; thus, at the time of investigation, all the cases had been recovered from the infection. So at the time of blood collection for laboratory confirmation, a maximum of them refused to give their blood sample for the test. The reason they gave was that now they are not suffering from the disease so they did not require tests anymore. Somehow, we manage to get five samples out of which one sample was found to be positive for varicella-zoster infection.

Varicella-zoster outbreak investigation at Bharno block of Gumla district

An outbreak was reported by the district response team in a village and a school located in that village. So, the observational visit was carried out at the school (Nav Utkramit Middle School) and the village where the outbreak occurred. Key interviews on the various aspects related to varicella/chickenpox infection were done with the people infected, their exposed contacts, their family members, and also with the service providers of that village who were directly or indirectly involved in the healthcare services in that area.

As per our investigation, the first case that fulfilled the case definition of varicella was a 12-year-old male student of class five who got the infection around mid of March 2022 (the student of Nav Utkramit Middle School, Atakcorakhas, Bharno). After the fifth day, his three brothers and five other students from his class got infected. The school was approximately 8 km from the CHC of Bharno, Gumla district. There was a continuous rise in cases in exposed families and neighbors in the village where the children reside. According to the case definition, 200 persons were found affected by the varicella-zoster/chickenpox infection and they belonged to 135 different households in that village. At the time of investigation, there were 21 active cases and the rest were either recovered or were still in the recovery phase in that village (Table 2).

**Table 2:** Outbreak investigation report at Bharno, Gumla district

Serial number	Age in years	Gender	Village	Date of onset of rash	Duration	Status (recovered or active)	Location of rash	Severity	Other symptoms	Vaccination status	Transmission setting	Household	Treatment taken
1	12 (index case)	M	Atakcorakhas	Around mid-March	15 days	Recovered	All over the body except the palm and sole	Yes	Fever, itching	Unknown	Unknown	1	No
2	9 (second case)	M	Atakcorakhas	7 days after exposure to the index case	7 days	Recovered	All over the body except the palm and sole	Yes	Fever, itching	Unknown	Home	1	No
3	7	M	Atakcorakhas	7 days after the index case	7 days	Recovered	All over the body except the palm, sole, and leg	Yes	Fever, itching	Unknown	Home	1	No
4	17	M	Atakcorakhas	17 days after the index case	7 days	Recovered	All over the body except the palm, sole, and leg	Yes	Fever, itching	Unknown	Home	1	No
5	9	M	Atakcorakhas	Last week of March	15 days	Recovered	All over the body	Yes	Fever, itching	Unknown	School	2	No
6	11	F	Atakcorakhas	1st week of April	14 days	Recovered	All over the body	Yes	Fever, itching	Unknown	School	2	No
7	13	F	Atakcorakhas	1st week of April	10 days	Recovered	All over the body	Yes	Fever, itching	Unknown	School	2	No
8	25	F	Atakcorakhas	Around mid-April	7 days	Recovered	All over the body except the palm and sole	Yes	Fever, itching	Unknown	Home	2	No
9	13	F	Atakcorakhas	7 days after the index case	15 days	Recovered	All over the body except the palm and sole	Yes	Fever, itching	Unknown	School	3	No
10	7	M	Atakcorakhas	Last week of March	7 days	Recovered	All over the body except the palm and sole	Yes	Fever, itching	Unknown	School	3	No
11	12	M	Atakcorakhas	7 days after the index case	15 Days	Recovered	All over the body except the palm and sole	Yes	Fever, itching	Unknown	School	4	No
12	10	M	Atakcorakhas	1st week of April	7 days	Recovered	All over the body except the palm and sole	Yes	Fever, itching	Unknown	School	4	No
13	7	M	Atakcorakhas	1st week of April	7 days	Recovered	All over the body except the palm and sole	Yes	Fever, itching	Unknown	School	4	No
14	11	F	Atakcorakhas	7 days after the index case	15 days	Recovered	All over the body except the palm and sole	Yes	Fever, itching	Unknown	School	5	No
15	8	M	Atakcorakhas	1st week of April	7 days	Recovered	All over the body except the palm and sole	Yes	Fever, itching	Unknown	School	5	No
16	6	M	Atakcorakhas	1st week of April	7 days	Recovered	All over the body except the palm and sole	Yes	Fever, itching	Unknown	School	5	No
17	12	F	Atakcorakhas	Last week of April	7 Days	Active	All over the body except the palm and sole	Yes	Fever, itching	Unknown	School	6	No
18	7	F	Atakcorakhas	3rd week of April	7 days	Under recovery phase	All over the body except the palm and sole	Yes	Fever, itching	Unknown	School	6	No
19	5	M	Atakcorakhas	3rd week of April	7 days	Under recovery phase	All over the body except the palm and sole	Yes	itching	Unknown	School	6	No
20	11	M	Atakcorakhas	Last week of March	15 days	Recovered	All over the body except the palm and sole	Yes	Fever, itching	Unknown	School	7	No
21	13	M	Atakcorakhas	15th April	7 days	Under recovery phase	All over the body except the palm and sole	Yes	No			8	No
22	7	F	Atakcorakhas	10th April	15 days	Recovered	All over the body except the palm and sole	Yes	Fever, itching	Unknown	School	9	No
23	9	M	Atakcorakhas	18th April	7 days	Under recovery phase	All over the body except the palm and sole	Yes	Fever, itching	Unknown	School	9	No
24	25	F	Atakcorakhas	18th April	7 days	Under recovery phase	All over the body except the palm and sole	Yes	Fever, itching	Unknown	Home	9	No
25	11	M	Atakcorakhas	15th April	10 days	Under recovery phase	All over the body except the palm and sole	Yes	Fever, itching	Unknown	School	10	No
26	14	F	Atakcorakhas	30th April	4 days	Active	All over the body except the palm and sole	Yes	Fever, itching	Unknown	School	10	No
27	9	F	Atakcorakhas	30th April	4 days	Active	All over the body except the palm and sole	Yes	Fever, itching	Unknown	School	10	No
28	2 months	M	Atakcorakhas	1st May	3 days	Active	On trunk and mouth	Yes	Fever, itching		Home	11	Yes
29	8	F	Atakcorakhas	1st week of April	15 days	Recovered	All over the body except the palm and sole	Yes	Fever, itching	Unknown	School	11	No
30	5	M	Atakcorakhas	Last week of April	7 days	Under recovery phase	All over the body except the palm and sole	Yes	Fever, itching	Unknown	Neighbor	12	Yes
31	20	F	Atakcorakhas	2nd May	2 days	Active	All over the body except the palm and sole	Yes	Fever, pain	Unknown	Home	13	No
32	28	M	Atakcorakhas	30th April	4 days	Active	All over the body except the palm and sole	yes	Fever, itching	Unknown	Home	13	No
33	5	F	Atakcorakhas	1st week of April	15 days	Recovered	All over the body except the palm and sole	Yes	Fever, itching	Unknown	Home	13	No
34	8	F	Atakcorakhas	30th April	4 days	Active	All over the body except the palm and sole	Yes	Fever, itching	Unknown	School	13	No
35	25	F	Atakcorakhas	28th April	7 days	Active	All over the body except the palm and sole	Yes	Fever, itching	Unknown	Home	13	No
36	2	M	Atakcorakhas	28th April	7 days	Active	All over the body except the palm and sole	Yes	Fever, itching	Unknown	Home	13	No
37	8	M	Atakcorakhas	2nd May	2 days	Active	All over the body except the palm and sole	Yes	Fever, itching	Unknown	Unknown	14	No
38	10	M	Atakcorakhas	2nd May	2 days	Active	All over the body except the palm and sole	Yes	Fever, itching	Unknown	Unknown	14	No
39	13	M	Atakcorakhas	29th April	6 days	Active	All over the body except the palm and sole	Yes	Fever, itching	Unknown	Unknown	15	No
40	3	F	Atakcorakhas	3rd May	1 day	Active	All over the body except the palm and sole	Yes	Fever, itching	Unknown	Neighbor	16	No
41	5	M	Atakcorakhas	1st week of April	15 days	Recovered	All over the body except the palm and sole	Yes	itching	Unknown	Home	17	No
42	13	M	Atakcorakhas	1st week of April	15 days	Recovered	All over the body except the palm and sole	Yes	Fever, itching	Unknown	School	17	No
43	16	F	Atakcorakhas	29th April	6 days	Active	All over the body except the palm and sole	Yes	Fever, itching	Unknown	Unknown	18	No
44	6	M	Atakcorakhas	28th April	7 days	Active	Hand, trunk	No	Fever, itching	Unknown	Unknown	19	No
45	6	F	Atakcorakhas	29th April	6 days	Active	All over the body except the palm and sole	Yes	Fever, itching	Unknown	Unknown	20	No
46	8	F	Atakcorakhas	4th May	1 day	Active (eruptive stage)	Trunk	No	No	Unknown	Home	20	No
47	11	F	Atakcorakhas	Last week of March	15 days	Recovered	All over the body except the palm and sole	Yes	Fever, itching	Unknown	School	21	No
48	8	F	Atakcorakhas	15th April	10 days	Recovered	All over the body except the palm and sole	Yes	Fever, itching	Unknown	School	21	No
49	18	M	Atakcorakhas	15th April	10 days	Recovered	All over the body except the palm and sole	Yes	Fever, itching	Unknown	Home	21	No
50	30	F	Atakcorakhas	25th April	7 days	Under recovery phase	All over the body except the palm and sole	Yes	Fever, itching	Unknown	Home	21	No
51	8	F	Atakcorakhas	Last week of April	7 days	Under recovery phase	All over the body except the palm and sole	Yes	Fever, itching	Unknown	School	22	No
52	5	M	Atakcorakhas	Last week of April	7 days	Under recovery phase	All over the body except the palm and sole	Yes	Fever, itching	Unknown	Home	22	No
53	10	F	Atakcorakhas	Last week of April	7 days	Under recovery phase	All over the body except the palm and sole	Yes	Fever, itching	Unknown	School	22	No
54	8	M	Atakcorakhas	1st May	3 days	Active	Trunk, face	No	Fever, itching, vomiting	Unknown	School	23	No
55	7	M	Atakcorakhas	1st week of April	15 days	Recovered	All over the body except the palm and sole	Yes	Fever, itching	Unknown	School	24	No
56	4	M	Atakcorakhas	3rd May	2 days	Active (eruptive stage)	Trunk, face		Fever, itching, cough	Unknown	Home	25	No
57	7	F	Atakcorakhas	Last week of March	15 days	Recovered	All over the body except the palm and sole	Yes	Fever, itching	Unknown	School	25	No
58	2	F	Atakcorakhas	Last week of March	15 days	Recovered	All over the body except the palm and sole	Yes	Fever, itching	Unknown	Home	25	No
59	10	F	Atakcorakhas	27th April	8 days	Active	All over the body except the palm and sole	Yes	Fever, itching	Unknown	School	26	No
60	8	F	Atakcorakhas	28th April	7 days	Active	All over the body except the palm and sole	Yes	Fever, itching	Unknown	School	26	No
61	25	F	Atakcorakhas	26th April	9 days	Active	All over the body except the palm and sole	Yes	Fever, itching	Unknown	Home	26	No
62	7	M	Atakcorakhas	15th April	19 days	Active	All over the body except the palm and sole	Yes	Fever, itching	Unknown	School	26	No

We interviewed the first case that was reported by the school to ANM and she reported to the district response team. The first case of that village was a 12-year-old male, an orphan boy who lived with his maternal aunt and four cousins. His rash first appeared around mid-March 2022 on the chest, and then back, followed by limbs and face. As per his information, the rashes were more than 50 in number. He did not seek any treatment from the nearest health center. His vaccination status was unknown. According to him, the duration of rashes was around 15 days. Along with rashes, he got on and off fever and itching all over the body. He was not suffering from any other diseases and was not on any immunosuppressive drugs. He could not recall any potential exposure to varicella-zoster infection in the weeks before the onset of rash, as he frequently visits his school for mid-day meals. The second case of that village was also among his two younger cousins and five classmates from his school. Family members of 17 households were infected with the same rashes along with itching and fever. A total of 61 persons were fulfilling the case definition of varicella infection and were infected after the index case (Table 2). The age group varied from two months to 30 years. None of them were vaccinated for varicella-zoster/chickenpox infection.

**Figure 2:**
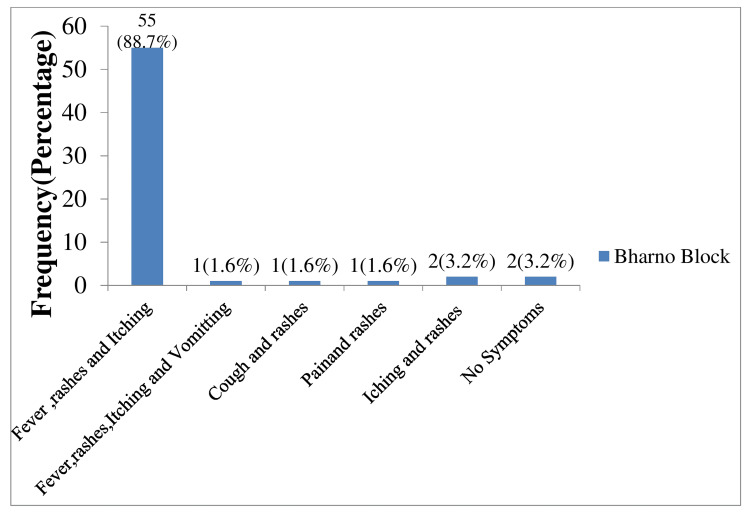
Clinical features among cases at Bharno block

Among all cases, macula-papular rashes occurred with itching all over the body. Most of them had rashes over the face, trunk, and limbs. One case (two months of age) reported maculopapular rashes on buccal mucosa with a history of 10 days of fever. Three people were found with few rashes. Out of 62 cases, 55 (88.7%) complained of fever, rashes, and itching, two (3.2%) complained of itching with rashes, one (1.6%) reported vomiting along with fever and rashes, one (1.6%) complained about pain and rashes, one (1.6%) complained of cough with rashes, and four (25%) complained of itching along with rashes (Figure 2).

Two people had a history of persistent fever with rigor for three to four days. Only one case reported fever with cough for one day. Two cases among the total cases visited the local faith healer for treatment during the illness. Most of the cases were applying ghee, goat milk, and coconut oil over the rashes and were using leaves of neem/Indian lilac to get relief from itching. One person was applying ragi (madua) flour over the rashes to get relief from itching.

None of the cases infected from the illness were suffering from any other diseases like asthma, cystic fibrosis, cancer, and HIV/AIDS. None of the cases were vaccinated with the varicella-zoster/chickenpox vaccine.

Since the first case was reported on mid of March and the incubation period of the varicella-zoster infection is an average of 14-16 days, thus at the time of investigation, we found that 28 cases had recovered, 11 were in the recovery phase, two were in active eruptive stage, and 21 cases were found active.

It is worth mentioning that rashes were also found among the animals of that village at the time of the investigation. Blood samples of 26 people were collected during the investigation, of which 20 were found to be positive for varicella-zoster infection.

Among the two outbreak sites, the attack rate was found to be 3.69% and the secondary attack rate was 60% at Bharno block, while at Itkhori block, the attack rate was found to be 0.4% and the secondary attack rate was found to be 25.8%. Gender-specific attack rate was found to be 0.38% for males and 0.42% for females at the Itkhori block, while at the Bharno block, the gender-specific rate was found to be 3.69% for both males and females (Table 3).

**Table 3:** Attack rate and secondary attack rate among both the sites

Block (district)	Vaccination	Population at risk	Cases	Attack rate	Attack rate for males	Attack rate for females	Secondary attack rate
Itkhori (Chatra)	0%	3996	16	0.4%	0.38%	0.42%	60%
Bharno (Gumla)	0%	1676	62	3.69%	3.69%	3.69%	25.8%

**Figure 3:**
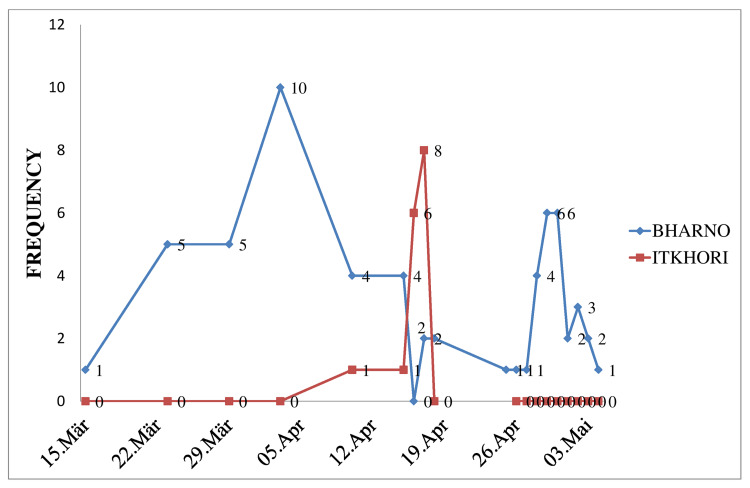
Epicurve showing the datewise number of cases for the onset of rashes at both the sites

The epidemic curve shows that at the Bharno block, the maximum number of cases were having an onset of rashes between the last week of March and the first week of April, whereas, at the Itkhori block, the maximum number of cases were having an onset of rashes in the mid of April (Figure 3).

Environmental assessment

Pitiz village of the Itkhori block was having a dense population. While at the Bharno block, investigations revealed that the primary case was a student who was responsible for this initial cluster as there was close interaction with other students of that school. At both the sites, the temperature was quite high, which was the potential for the source of varicella-zoster infection. We also discovered the presence of pox-like lesions among the pet animals like cows, buffaloes, and goats residing at the Bharno block.

## Discussion

The present investigations describe the outbreak of the chickenpox epidemic that has been confirmed in the tribal area of Jharkhand, India (Atakcorakhas village, Bharno block in Gumla and Pitiz village, Itkhori block in Chatra) using the serological method. The first incidence was reported in mid-March in Atakcorakhas and on April 10th, 2022 in Pitiz village. Similar findings were found in studies done by Pall et al. and Malakar et al. where they also found the highest number of cases in March and April [13,14]. Interference with the transmission of VZV by high temperature and in dry seasons has been suggested as a possible reason for this seasonal variation [15,16]. In the Atakcorakhas village of Bharno block, the number of chickenpox cases was higher among school-aged children than among adults. It has been seen that in the districts having low measles vaccination coverage (<80%), during outbreak settings, the majority of cases were among the children aged less than 10 years [17], whereas adults were found to be affected more than children in the Pitiz village. This may be due to the nonexposure of adults with VZV during their childhood. Despite different attack rates of 3.69% and 0.4% in Atakcorakhas and Pitiz villages, respectively, female cases were greater than male cases in both the villages. According to Malakar et al. and Chakraborty et al., people refuse to take treatment for chickenpox and prefer to utilize solely their traditional cures, and this was also noted in our research where the team had difficulty collecting specimens in Pitiz village due to patient refusals. A total of 31 samples were collected from both villages [14,18].

Out of 31 samples, a total of 21 were found to be positive for varicella-zoster infection using the ELISA method. This could be due to the fact that IgM antibodies are typically detectable one to two weeks after the initial infection. All of the patients had a history of contact with a case of chickenpox, but none of them had ever been vaccinated against VZV. The vast majority of cases recovered on their own, with no serious difficulties or complications.

At the time of investigations, in Pitiz village, all the cases had been recovered from the infection and the source of infection was not clear. While in Atakcorakhas, close interaction in school was one of the contributing factors to the clustering of chickenpox infections. During our investigations, it came to light that the medical officer of Bharno block organized an outpatient camp in the affected sites, and suspected cases were advised to be isolated to decrease disease morbidity, and symptomatic treatment for clinical cases was provided. Local neighbors were informed about the disease's route of transmission, and precautionary measures have also been proposed to them. The team went to a local school and houses of cases, where they spoke with the cases and the afflicted area's staff. The team examined the entire school campus for sanitation, hygiene, and house isolation. Low literacy rates, inadequate air ventilation, and sanitary conditions were all found to be likely causes of illness and its transmission.

Following verification, it was determined that there was no delay in reporting index cases to higher authorities, but that due to village myths, communities failed to take control measures at the proper time, resulting in the disease spreading swiftly at both the sites. Health workers like accredited social health activists (ASHA) and ANMs visited the households in their assigned areas on a regular basis, they educated the people about the disease's threshold and the significance of timely reporting, even though the villagers refused to offer their samples. At Bharno block, students continued to come to the school for mid-day meals even after infection, which resulted in the rise of cases in that area. Similar findings were found by Dworkin et al. and Galil et al. where the outbreak of chickenpox was found more among the students of institutions and schools [19,20].

Finally, this inquiry presents in-depth information about the chickenpox outbreak in Jharkhand, India. Since chickenpox is a vaccine-preventable disease and infection in children is associated with higher morbidity, at least in the northern part of India, in a tribal state of India like Jharkhand, it is suggested that varicella vaccination should be considered in the routine immunization program, as people of the state are underprivileged. The two-dose strategy for the measles vaccine has already started in four states and union territories in India [16-21].

## Conclusions

Most of the cases were self-limiting and recovered at the Itkhori block, while at the Bharno block, there were 20 active cases and the rest were either recovered or were still in the recovery phase. Chickenpox is still widespread in Northern parts of India like Jharkhand. Therefore, it is recommended that future varicella outbreaks should be prevented by establishing and maintaining continuous varicella surveillance, reporting, and preparing an appropriate response when a case of varicella is identified in any particular setting, and notifying and identifying contacts without evidence of immunity and providing appropriate prophylaxis as indicated. If possible, the contacts exposed to the index case should be vaccinated within two to three days to control the spread of the virus.

Primary care physicians and grassroots level workers might play a critical role in providing accurate and timely information to health authorities, allowing for early intervention and outbreak management. In addition, the study emphasizes the importance of investigating further chickenpox outbreaks in India's tribal and other populations.
